# Gold Nanoparticle Contrast Agents in Advanced X-ray Imaging Technologies

**DOI:** 10.3390/molecules18055858

**Published:** 2013-05-17

**Authors:** Sungsook Ahn, Sung Yong Jung, Sang Joon Lee

**Affiliations:** 1 Biofluid and Biomimic Research Center, Pohang University of Science and Technology, Pohang 790-784, Korea; 2 Department of Mechanical Engineering, Pohang University of Science and Technology, Pohang 790-784, Korea

**Keywords:** gold nanoparticles (AuNPs), X-ray imaging, contrast agent

## Abstract

Recently, there has been significant progress in the field of soft- and hard-X-ray imaging for a wide range of applications, both technically and scientifically, via developments in sources, optics and imaging methodologies. While one community is pursuing extensive applications of available X-ray tools, others are investigating improvements in techniques, including new optics, higher spatial resolutions and brighter compact sources. For increased image quality and more exquisite investigation on characteristic biological phenomena, contrast agents have been employed extensively in imaging technologies. Heavy metal nanoparticles are excellent absorbers of X-rays and can offer excellent improvements in medical diagnosis and X-ray imaging. In this context, the role of gold (Au) is important for advanced X-ray imaging applications. Au has a long-history in a wide range of medical applications and exhibits characteristic interactions with X-rays. Therefore, Au can offer a particular advantage as a tracer and a contrast enhancer in X-ray imaging technologies by sensing the variation in X-ray attenuation in a given sample volume. This review summarizes basic understanding on X-ray imaging from device set-up to technologies. Then this review covers recent studies in the development of X-ray imaging techniques utilizing gold nanoparticles (AuNPs) and their relevant applications, including two- and three-dimensional biological imaging, dynamical processes in a living system, single cell-based imaging and quantitative analysis of circulatory systems and so on. In addition to conventional medical applications, various novel research areas have been developed and are expected to be further developed through AuNP-based X-ray imaging technologies.

## 1. Introduction

Colloidal gold (Au) was discovered by scientists as early as in the fourth century B.C. Since then, colloidal Au solutions have been employed particularly for medical purposes. The first intravenous injection of Au solution was performed in 1880 to treat alcoholism [1], and later for inoperable cancer patients [2]. Au solution is also used to trigger specific biomolecular interactions in spinal-fluid and blood-serum proteins [3]. On the other hand, isotope ^198^Au (half-life = 65 h) is employed in cancer care facilities for therapeutic use [4]. Recent applications of colloidal Au are more versatile, including catalytic processes and electron transport in biomacromolecules [5], drug/imaging agent transport into cells by endocytosis pathway [6], investigation of cell motility [7], improvement of PCR efficiency [8], and so on. Biomedical use of gold nanoparticles (AuNPs) was recently reviewed by Dykman *et al.* [9], where diagnosis, therapy, drug carrier and immunological properties of AuNPs are summarized as major applications. 

One of the advantageous physical properties of Au is its high X-ray absorption when employed in X-ray imaging. Since its discovery by Röntgen in 1896 [10], X-ray imaging has expanded its unique territory in biomedical applications as one of the popular tools to see through a body nondestructively. X-rays have been developed for targeted researches and medical uses depending on the type of the light sources and generated energy regions. In this point, AuNP-based X-ray imaging can provide a great potential for broad scopes of yet-reported future researches and applications. In this review, AuNP-based X-ray imaging technologies and their applications are discussed. First, X-ray imaging is investigated fundamentally from a technological point of view. X-ray imaging equipment set-up, source types and technologies are summarized. This explains the suitability and possible improvements in the X-ray imaging technologies. Second, contrast principles and general contrast agents in X-ray imaging are discussed. Third, AuNP-based X-ray imaging is investigated with representative applications reported in recent researches. In addition to medical applications, AuNP-based X-ray imaging is highlighted as one of the pioneering tools for cutting-edge research in broad scientific areas.

## 2. X-ray Imaging Devices

### 2.1. X-ray Sources

*X-ray tubes* generate X-rays based on the classical Röntgen mechanism, which is the common source for medical, dental and routine industrial uses. Electrons emitted from a heated tungsten filament (cathode) are accelerated to 20–100 keV and electrostatically focused onto a metal anode. The electrons lose energy as they interact with the anode and some of this energy is emitted in the form of X-rays. The source size is typically a few mm, but that size can be reduced to a few μm with a smaller electron filament and more sophisticated focusing. The emitted X-rays are both characteristic (fluorescent X-rays from the target material) with discontinuously defined energies and bremsstrahlung X-rays with a continuous energy spectrum. Although this conventional source provides a large field-of-view usually required by many medical applications, the source performance is limited by low resolution and absorption-only capability, thus being insensitive to subtle changes in electron density in biological tissues. 

High-energy (80 MeV) *synchrotron sources* first became available in the 1940s [11]. The accelerator-based strategy results in small source size, small angular divergence and high power sources, which cannot be provided by the standard Röntgen mechanism, even though they are required for novel radiology. In addition, compared with those generated by X-ray tubes, synchrotron X-rays possess considerably higher energy, wider X-ray spectra, excellent collimation, and linear polarization [12]. Accelerator technology enables electrons to act as free particles. The cross-section of an electron-beam can be controlled with an accuracy better than 1/1,000 of a mm over a several-hundred-meter circumference [13]. Electrons accelerated to high energies (several GeV) radiate a copious amount of X-rays when they pass through magnets. Since these electrons move at a speed of the light, the emitted X-rays are highly collimated. The electron beams are maintained in near-circular orbits through a ring of bending magnets. The X-ray beams derived from these magnets have a continuous spectrum useful for high-resolution spectroscopy. The high coherence also enables phase contrast. Over the last few decades, dozens of synchrotron light sources have been built at major national laboratories around the world. Recently, microfocus X-ray sources have been developed by combining the high performance of a synchrotron radiation source and the easy use of an X-ray tube, which results in high-resolution X-ray images [14,15,16]. 

### 2.2. X-ray Condenser

Most X-ray applications in medicine, dentistry, routine industrial testing, and crystallography do not use any optical elements. Simple lenses and mirrors are not useful in X-ray technologies because the real part of the refractive index of most materials for X-ray is close to 1.0. In addition to the development of X-ray sources, advances in X-ray optics such as compound refractive and zone plate lenses enable nano-scale imaging [17,18,19]. Even though these modern X-ray optical devices are prerequisites for high-resolution X-ray microscopes and microprobes, they have become available only recently. *Zone plates* are circular diffraction gratings with a radially increasing line density. The spacing is arranged to primarily focus first-order diffracted waves. The zone boundaries (*r*_n_) are related by the expression, *r*_n_^2^ = *nf*λ, where *n* is the zone number, *f* is the focal length, and λ is the wavelength. For 50 nm resolution, the thickness of the zone-plate needs to be in the order of a μm. The fabrication of such narrow structures is a difficult challenge. *Compound refractive lenses* are another X-ray condenser. Concave lenses focus, whereas convex ones defocus. Given that the real part of the refractive index of materials with X-ray is close to (but slightly less than) 1.0, the focal length of the most highly curved lenses is measured in miles. Although single lenses are relatively useless, a combined focusing effect using dozens of lenses in a row enables reasonable focal lengths. But a major limitation is the absorption of X-rays in the lens material. To minimize the absorption, the lens must be made from a material such as aluminium, beryllium, or lithium. The advantage of compound refractive lenses is that they are much more robust than zone plates, and suitable for focusing high-energy beams. *Kirkpatrick-Baez mirrors* focus X-rays at grazing incidence by a complex multilayer coating. In grazing incidence, the reflectivity is close to 100% if the grazing angle is within the range of total external reflection. *Monocapillary optics* is particularly useful in X-ray microscopes, microprobes, and similar devices. By carefully shaping the interior surface of the glass capillary to a paraboloidal or ellipsoidal shape, a collimated X-ray beam can be focused by only using a single grazing incidence reflection. 

### 2.3. X-ray Detector

*Photographic film* is an old-fashioned X-ray detector still used in medicine and dentistry. It is relatively inexpensive and provides a permanent record with a spatial resolution typically ranging within 10–100 μm. Photographic film is developed using wet chemistry in a film-developing apparatus. *Image plates* are similar to film in resolution and sensitivity, but they are reusable. *Image intensifiers* use a phosphor screen to convert X-rays to visible light, followed by a photocathode that converts visible light to electrons. Image intensifiers possess excellent time resolution, but the output is somewhat noisy and unsuitable for quantitative measurements. *Charge coupled device* (*CCD) detectors* are widely used in digital photography with scintillator crystals. The pixel size is typically 10–20 μm. CCDs can directly detect X-rays, but they can also suffer radiation damage at high radiation doses. To protect a CCD from damage or to image a large field-of-view, X-rays are first converted into visible light in a scintillator screen, and then the visible light image is transferred onto the CCD. *Energy dispersive detectors* measure the X-ray energy, whereas the aforementioned detectors record the X-ray images (radiographs). Lithium-drifted silicon or germanium detectors provide no positional information, but they measure the X-ray energy with sufficient accuracy (100–200 eV), which is useful to identify characteristic X-rays in a fluorescence spectrum. Basically, the X-ray energy is converted to electron-hole pairs (3.7 eV for one pair in silicon), creating a tiny current pulse. The total charge in the pulse is then measured. For the highest accuracy, the detector is cooled and often used with a multichannel analyzer.

## 3. X-ray Imaging Technologies

Visible light microscopy is convenient and ubiquitous. However, the resolution is limited by the wavelength and many materials are opaque to visible light. In other imaging methods, such as transmission electron microscopy (TEM), scanning electron microscopy (SEM) and scanning probe microscopy (SPM), ultra-thin samples need to be prepared and/or surface-only information is obtained. In the 1950s, Newberry produced a shadow X-ray microscopy (XRM) in which the specimen is placed between the source and a target plate. This became the basis for the first commercial XRM. XRM utilizes electromagnetic radiation of the X-ray which do not easily reflect or refract, and are invisible to the human eye. XRM is basically a contrast imaging technology exploiting the differences in the X-ray absorption. The basic process of an XRM is to detect X-rays that pass through a specimen by exposing film or a CCD detector connected with scintillator crystal to convert X-ray into visible light. 

The resolution of XRM lies between those of optical and the electron microscope, but, XRM is advantageous over conventional electron microscopy because biological samples can be imaged in their *in situ* state. Electron microscopy is widely used to obtain images with nm-level resolution but relatively thick living cell cannot be observed because of their penetrating ability is less effective than that of X-rays. Given the highly penetrating property and short wavelength of X-rays, *in situ* tomographic three-dimensional (3D) information are also obtained by XRM, even for samples too large for electron microscopy. In addition, the resolution of XRM is better than that of visible light microscopy. Diverse XRMs with various fields-of-view (from cm to μm) and resolutions (from tens of μm to nm) are available. A commercially available nano-scale XRM utilizes synchrotron-based X-ray optics and provides sub-100 nm 3D volumetric resolution [16]. X-ray analytical techniques such as X-ray fluorescence, spectroscopy and diffraction enable resolutions down to nm-scale. These XRMs are applied to various life science studies such as cell adhesion and cell distribution in 3D porous composite scaffolds [20,21]. The Advanced Light Source in Berkeley holds the world record for spatial resolution down to 15 nm obtained with Fresnel zone plates, and can combine a high spatial resolution with a sub-100 ps time resolution [22].

Table 1 compares the various types of microscopy technologies and their properties [23]. *In projection* *XRM*, magnification is achieved by positioning the sample close to a point-source of X-rays. A magnified projection image of the object is formed on the detector with a magnification equal to the ratio of the source-detector and source-object distances. By rotating a sample, a series of projection images at different angle is acquired, for which tomographic reconstruction algorithm is applied to obtain the whole internal 3D structure. The resolution of projection-based XRM is limited by the size of the X-ray source and the resolution of the X-ray detector (a commercially available value is approximately 1 μm). 

**Table 1:** Microscopy technologies and their properties [23]. Adapted by permission from Xradia Inc.

Microscopy Type	Detection	Resolution	Contrast
Visible light	Transmitted light	500 nm	Bright field
		500 nm	Phase contrast
	Scattered light	500 nm	Dark field
	Fluorescence	50–500 nm	Label
Transmission electron microscope (TEM)	Scattered electrons	0.1–1 nm	Heavy metal stain
Scanning electron microscopy (SEM)	Secondary electrons	3–10 nm	Surface relief
Scanning tunneling microscopy (STM)	Tunneling current	0.1 nm	Surface atoms
Atomic force microscopy (AFM)	Force on probe tip	0.5 nm	Surface relief
X-ray projection microscopy	Transmitted X-rays	> 1000 nm	Absorption
Transmission X-ray microscopy (TXM)	Transmitted X-rays	25 nm	Absorption
		25 nm	Phase contrast
Scanning Transmission X-ray microscopy (STXM)	Transmitted X-rays	25 nm	Absorption
			XANES (chemical)
Nanoprobe microscopy	Fluorescence	30 nm	Elements
	Diffraction	30 nm	Strain

In *lens-based XRM*, high resolution is attained by an optical set-up similar to standard light microscopy. The X-rays emitted by the source are concentrated onto the sample by a condenser lens, and the transmitted X-rays are imaged onto a detector by an objective lens. Currently, 3D XRM utilizing high resolution zone plate lenses are available to achieve a resolution below 50 nm. For phase-contrast imaging, an Au phase ring is inserted at the back focal plane of the objective lens. The use of a capillary condenser lens to focus X-rays from the X-ray source onto the sample provides high throughput and short exposure time. In *scanning* *XRM*, a zone plate lens is used to focus the X-ray beam to a fine spot, through which the specimen is raster-scanned. Transmitted, fluorescent or diffracted X-rays are detected at each scan position, thereby it maps out the chemical, elemental or crystallographic phase properties of the specimen. Considering the requirements for high-intensity X-ray beams, the use of scanning X-ray microscopes is limited to synchrotron radiation laboratories.

## 4. Contrast in X-ray Imaging

Since its discovery, medical radiology has been one of the most popular applications of X-rays. In most cases, the contrast in radiology images is based on the different X-ray absorption of the different parts of a specimen. However, absorption is actually limited for X-rays. Weak absorption means small absorption differences among the tested materials, resulting in limited contrast thus low image quality. When an X-ray beam traverses a matter both absorption and deflection of photons occur [24]. The intensity reduction caused by these processes defines the degree of X-ray attenuation, which obeys the Beer-Lambert law: *I* = *I*_0_e^−*μx*^, where *I* is the transmitted X-ray intensity, *I*_0_ is the incident intensity, and *x* is the thickness of the matter. The mass attenuation coefficient (*μ*) is the sum of the three interactions between X-ray photons and traversed matter in the proper energy range for diagnostic imaging by the unit of cm^2^/g: coherent scattering (*ω*), the photoelectron effect (*τ*), and Compton scattering (*δ*): *μ* = *ω+* *τ + δ*. Coherent scatterinδg (*ω*) produces scattered radiation and noise on X-ray films, but its effect on X-ray image quality is minor. But the photoelectron effect (*τ*) is considerable when the X-ray photon energy is greater or almost the same as the electronic binding energy. The greater the photon energy, the less X-ray absorption will occur in this process, thus *τ* is inversely proportional to the third power of photon energy *E*: *τ* ∝1/*E*^3^. Compton scattering is responsible for almost all scattered radiation, which both increases noise and decreases contrast. The quantity of Compton scattering diminishes as the X-ray photon energy increases, so that high energy photons are more likely to pass through the human body than low-energy photons. As a result, the radiation exposure to patients is lower with high energy X-rays than that with low-energy X-rays. Since very low energy X-rays will produce unacceptable radiation doses to patients and high-energy X-rays diminish the inherent contrast, X-ray energies used in current medical imaging procedures represent a compromise between optimal image quality and patient radiation dose. The inherent contrast between bone and other tissues is large enough for clinical use in the low to middle X-ray energy range.

The mass attenuation coefficient increases with atomic number increase of elements in periodic table, and decrease with energy increase of X-rays [25]. Many theoretical and experimental studies have also shown that higher atomic number elements demonstrate superior X-ray attenuation ability at normal or even higher operating tube voltages due to the higher K-edges of heavy elements. The K-edges of heavy elements lies within the diagnostic X-ray spectrum as such an abrupt increase in attenuation at discrete energies near the K edge is observed. Thus a contrast media based on elements with higher atomic number will be more advantageous in terms of intrinsic contrast, lower dose requirement and lower radiation exposure to patients. To increase X-ray contrast, long-time exposure and relatively high doses also can be attempted, which are not advisable for medical application though. Instead, the contrast can be increased by injecting a high-contrast material into the imaged specimen. Angiographic radiological examinations are commonly applied to ongoing pathologies. Currently, systematic screening of breast cancer by mammography is still bothered because of weak contrast. X-ray doses for enhanced contrast are actually unacceptable for a large portion of the female populations, even though very early breast cancer detection leads to almost 100% successful disease eradication [26].

### 4.1. Absorption-Contrast X-ray Imaging

Absorption-contrast X-ray imaging visualizes the X-ray attenuation variation within the volume of a given sample, whereas phase-contrast X-ray imaging visualizes spatial variations in X-ray refractive indices. Absorption-based X-ray imaging is an important paradigm of X-ray radiography, and it enables a vast domain of applications, such as imaging of bone fractures [27], coronary arteries [28] and the diagnosis of breast cancer [29]. All these applications utilize the high penetrating nature of X-rays. In absorption contrast, the shadow cast by the object is softened at the edges by a mechanism known as penumbral blurring (e.g., source-size blurring) [30]. The detrimental effects of such blurring can be reduced by making the object-to-detector distance or the source size as small as practicable. The former used in conventional radiography works by placing the imaging detector close to the sample to be imaged. This simple and powerful technique makes contact-mode absorption-contrast radiography insensitive to the refractive effects of a typical sample. Different from synchrotron sources, in conventional medical X-ray imaging, the source size is too large for phase contrast and there is a limitation in increasing the distance between the sample and the detector. 

**Figure 1:**
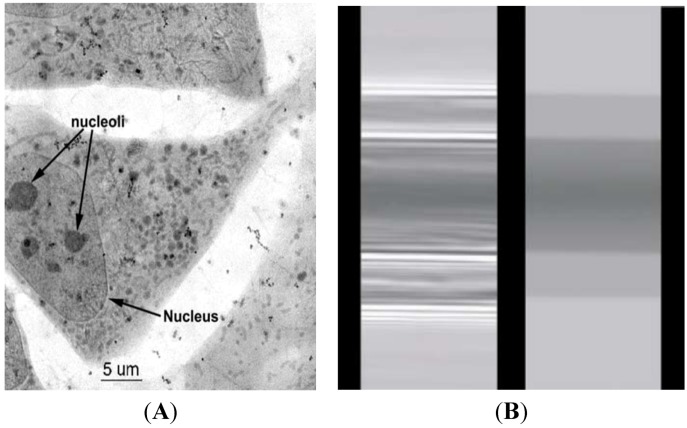
(**A**) Cryo X-ray microscopy of 3T3 cells [31]. (**B**) Direct comparison between the phase-contrast radiological image based on coherence of an optic fiber (on the left) and the corresponding absorption-contrast image (right) [34]. Adapted by permission from John Wiley and Sons and IOP Publishing.

In the X-ray imaging applications, the contrast effect can facilitate tracking of water and bubble locations as well as air-water interfaces in the samples [31,32,33]. By these methods, the water flow inside the xylem vessels of a plant is advantageously tracked to understand the water flow mechanism in the plant systems. *Water window* is a contrast imaging technology that uses the absorption difference between the carbon and the oxygen in soft X-rays. The water window covers the soft X-rays between the K-absorption edges of oxygen (the main element for water) at a wavelength of 2.34 nm and carbon (the main element of living cells) at 4.4 nm. This corresponds to X-ray energies of 280 and 530 eV, respectively. Water is transparent to these X-rays, whereas nitrogen and other elements in biological specimens are mainly absorptive. These wavelengths are used in an X-ray microscope to observe living specimens [26,33]. X-ray image contrast generated by water benefits many biological systems. Mouse 3T3 cells rapidly frozen and viewed on a cryo-stage by an XRM, reveal superior morphological preservation [31]. The cell is initially living and then examined under liquid nitrogen temperature in the cryo-stage. No chemical fixatives or contrast enhancement agents are used. Images are obtained using a photon energy of 517 eV (λ = 2.4 nm), X-ray magnification of 2400×, and 20 nm pixel size. Image size in pixels is 2035 × 2033. As shown in Figure 1A, numerous organelles, granules of various sizes, and tubular structures (presumably mitochondria) in the cytoplasm can be observed by XRM. Even the thickest region of the cell, the nucleus (approximately 5-nm thick in these cells) is visualized. Several nucleoli are clearly visible within the nucleus, and sharp images of the surrounding nuclear membrane are also obtained.

### 4.2. Phase-Contrast X-ray Imaging

Absorption is not the only type of interaction between X-rays and matters that can enhance the contrast in radiological images. X-ray sources with reasonably high coherence (such as synchrotron) are required for phase-contrast. For example, Figure 1B shows the difference between a phase-contrast radiological image based on coherence-enhanced imaging at the left, and the corresponding absorption-contrast image at the right. A divergent and non-coherent source (left) produces an absorption-contrast image, whereas a collimated and coherent source (right) produces edge enhancement. Without increasing the X-ray dose, the visibility of the object becomes better with phase contrast [34]. Therefore, the dose can be substantially reduced without jeopardizing the practical image quality. Although phase-contrast radiology depends on the coherence of X-rays from synchrotron sources, real-time studies with high spatial resolution are still difficult. This problem has been tried to be solved using unmonochromatized synchrotron X-rays [34].

The three principal interactions of X-rays with matters are absorption, refraction, and scattering [35]. Conventional radiography uses X-ray absorption to achieve contrast, but the samples also refract and scatter photons. The refracted and scattered photons create noises in the image and reduce edge definition. By comparison, diffraction-enhanced imaging (DEI) separately measures refracted X-ray photons from non-refracted ones. Density differences in a sample result in X-ray refraction. In other words, DEI allows observations on the boundaries between tissues having different densities. Minimal X-ray absorption is possible if high-energy X-rays are used (30–40 keV), thereby resulting in low radiation damage. For example, by a non-destructive DEI technique using synchrotron X-rays, the morphology of live plant seeds is continuously observed for novel insights into the developmental processes of plant anatomy [35]. 

In conjunction with the absorption-based imaging, phase-contrast can provide structural details of biological systems. Although most nanoparticle (NP) designed as contrast enhancer in the imaging utilize absorption-based mechanism, effective phase-contrast needs to be combined for more information. Typically, there is a trade-off between the maximized absorption-contrast and maximized phase-contrast, because absorption-contrast is maximized at the sample-detector contact mode. Therefore, from a technological point of view, the optimized sample-to-detector distances needs to be obtained experimentally. 

## 5. Contrast Agents in X-ray Imaging

Apart from modulating X-ray properties and developing optical set-up, contrast agents are employed in numerous applications for enhanced image quality. In biomedical studies, X-ray imaging has advantageously utilized contrast agents typically similar to and characteristically different from other biomedical imaging modalities. In this review, gas form contrast agents and iodinated derivates are mainly compared with gold nanoparticles (AuNPs) due to their unique applicability and popularity in medical use. Many contrast agents have been and are to be developed further. However, a variety of candidate materials for X-ray contrast enhancer remains in open possibilities due to limited spaces. 

### 5.1. Gas Form Contrast Agents

**Figure 2:**
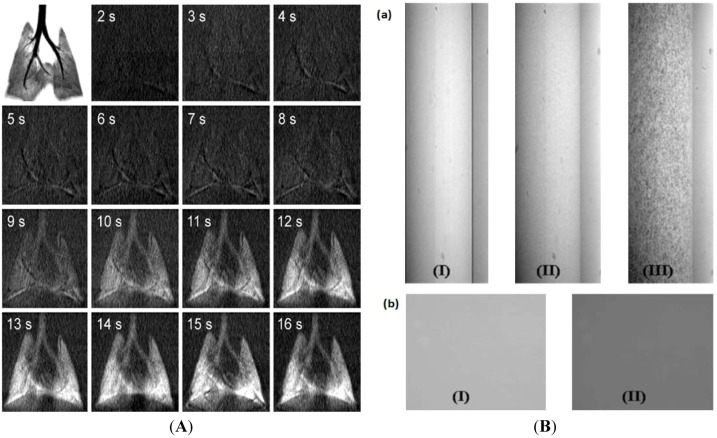
(**A**) A dynamic flat-panel volumetric preclinical micro-CT scanner (eXplore Ultra, GE Healthcare, London, ON, Canada) images of rat lungs. Top left frame shows a minimum intensity projection of an oxygen-filled rat lung. Subsequent frames show the evolution of Xe with baseline image subtracted at 1s intervals. The field-of-view for each frame is 36.5 mm × 36.5 mm [36] (**B**) (**a**) Typical X-ray images of an (I) empty tube (II) blood flow, and (III) blood flow seeded with Iopamidol-incorporated microparticle inside the tube. (**b**) Comparison of absolute X-ray absorption of (I) stationary pure blood and (II) blood mixed with pure iopamidol. Images are obtained by 3^rd^ generation synchrotron X-ray source with unmonochromated beamline (bending magnet; energy range of 4–15 keV) [40]. Adapted by permission from the American Physiological Society and Elsevier.

Gas phase contrast agents are uniquely utilized in X-ray imaging. The tracking pathway obtained by gas form contrast agents cannot be replaced by other solid or liquid phase contrast agents. Ventilation flow is quantified according to the dynamic contrast variation in the in X-ray computed tomography (X-ray CT) image, with the aid of inhaled Xe-contrast agents [36]. The delivery of Xe to the lung is analogous to the intravenous seeding of contrast agents for quantitative blood flow measurements. The non-invasive imaging of ventilation flow in the lung (mL/s) can reveal the symptoms of various lung diseases in humans, including chronic obstructive pulmonary diseases. Continuous CT image acquisition by Xe-contrast enables ventilation measurements in rat lung *in vivo* with low voxel noise. Time-resolved X-ray images are consecutively obtained (Figure 2A), which demonstrates the inhalation of Xe in rats during the consecutive 16 s at 1 s interval, demonstrating an increase in Xe inhalation during the breathing cycle. 

### 5.2. Iodine-Incorporated Particles

Iodinated derivative is one of the most common contrast enhancers for X-ray imaging. They are small in moelcular weight (approximately 800 to 1,600 Da) and water-soluble. The monomeric ionic agent is diatrizoate, while the monomeric non-ionic agent is iopromide. The dimeric non-ionic compounds are iotrolan and ioxaglate, which exhibit high X-ray absorption due to their high iodine contents [37]. The main difference of these iodinated contrast agents is osmolality: Ionic monomers exhibit high osmolality (1500–2300 mOsm/kg) at clinically relevant concentrations. Non-ionic monomers and ionic dimers are slightly hypertonic (500–800 mOsm/kg). Non-ionic dimers are isotonic to blood (approximately 300 mOsm/kg). Osmolality affects not only the tolerability of the unencapsulated agents but also their applicability for the entrapment in carriers [38]. Recently, osmolality of iodinated contrast media has been overcome by developing iso-osmolar media and various technologies for encapsulation of Iodinated contrast media [39]. 

After intravenous injection, these extracellular contrast agents briefly stay in the blood stream and then diffuse to the extracellular spaces of all non-neural tissues followed by excretion through the kidneys [41]. Iodine contrast agent-carrying liposomes are used for the improved detection of tumors, inflammations and infections [42]. These agents can detect small foci of metastatic cells, especially in lymph nodes and *in vivo* histological characterization of such lesions [43]. Iodinated nanoparticles designed to be used as a local drug delivery system [44]. The advantage of employing the radiopaque nanoparticles is tracking the diffusion of the drug-loaded nanoparticles in the body after a local administration. As an example, the diffusion of an anticancer drug loaded inside the iodinated nanoparticles could be monitored after an intratumoral injection [44]. Iodinated biodegradable polymeric blood pool contrast agent able to induce an exploitable contrast enhancement. The main advantage of polymeric system compared to lipid ones, lies in their stability and handling [45].CT scan would require the encapsulation of high contrast agents in a relatively small carrier for effective targeting and contrast enhancement in tissues (*i.e.*, density increase » 60 HU). Although an advanced multi-detector of CT can improve spatial and temporal resolution, CT still requires 10 times higher concentrations of contrast agents than MRI to achieve useful images (1 mg iodine/g tissue = 25–30 HU). Synchrotron sources have become more popular than conventional X-ray for tomographic imaging at μm-scale resolution [46], but they still need a high dose of contrast agent. Conventional iodine-based contrast agents dispersed in water are widely used as a solution-type X-ray contrast agent [46,47]. 

In most medical applications, liquefied contrast agents flow through the blood vessels during the expected life-time. However, individual flow tracers need to be detected discontinuously to quantify biofluid flows for velocimetric information. In this concept, solidified iodine-based flow tracing sensors are designed by encapsulating an iodine contrast agent (iopamidol) into the cross-linked polymeric microparticle [40,48]. Figure 2B(a) shows the X-ray images of an empty tube (I), pure blood flow without any flow tracer (II), and blood flow mixed with iopamidol-incorporated polymeric microparticles (III). The X-ray image of pure blood flow (II) is enhanced only by the diffraction contrast at the edges of red blood cells (RBCs). On the other hand, when blood is mixed with Iopamidol-incorporated polymeric microparticles (III), the X-ray image contrast of blood flow is enhanced by the diffraction of a cell as well as the enhanced absorption contrast by the introduced microparticles. In Figure 2B(b), the X-ray images of pure blood (I) and iopamidol-mixed blood (II) are compared in the stationary state. Iopamidol-mixed blood shows a darker image than pure blood because of enhanced X-ray absorption by iopamidol. However, different from iopmidol-incorporating microparticles, pure iopamidol in blood cannot provide velocimetric flow information because of the lack of discontinuous contrasts. The X-ray images of a mouse artery are captured where the absorption contrast also plays an important role even if the refraction effect is dominant [49]. 

### 5.3. Gold Particles as a New Contrast Agent in X-ray Imaging

Over the last three decades, no fundamental improvement in clinical X-ray contrast agents has been reported. The iodine-based chemical platform has not been replaced, despite its serious limitations in medical imaging, including short imaging time, the need of catheterization in many cases, occasional renal toxicity, and poor contrast in large patients. Gold nanoparticles (AuNPs) have been introduced to overcome this limitation [50]. The side effects of several iodine agents are caused by high osmolality: Iodine agents only contain three (monomer) or six (dimer) iodine atoms per molecule. By contrast, 1.9 nm diameter spherical AuNPs contain approximately 250 Au atoms [50]. Thus, AuNPs have a negligible osmolality of 7.2 mM, to which saline can be added more to achieve iso-osmolality. The low viscosity of AuNP solutions is also another advantageous property for intravenous injections. Au has remarkable advantages as an effective X-ray contrast agent when applied in various biofluids. Au has a higher absorption coefficient than iodine, and experiences less interference of bones and tissues, resulting in improved contrast at lower doses. Compared with low-molecular-weight iodine solutions, NPs can remain in the blood longer, benefiting prolonged imaging time. 

**Figure 3:**
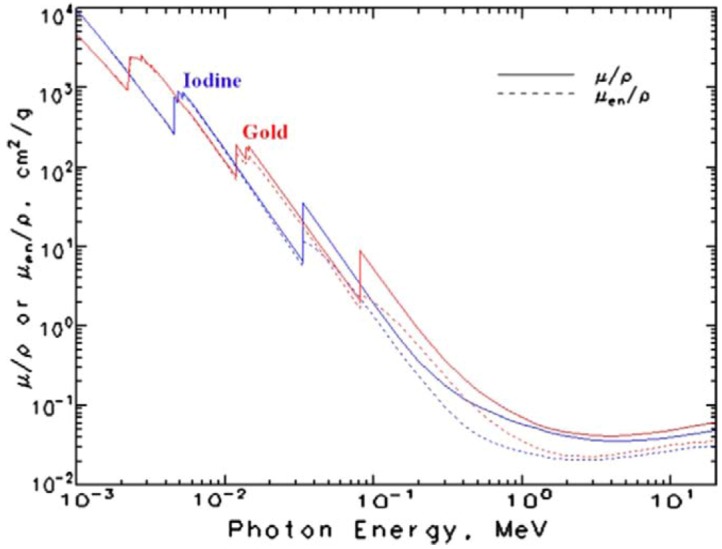
Energy-dependent X-ray absorption coefficients of iodine (I) and gold (Au) [51]. Adapted by permission from National Institute of Standards and Technology (NIST).

In the controlled energy region shown in Figure 3, the X-ray absorption efficiency of Au (5.16 cm^2^/g at 100 keV) is much higher than that of iodinated agents (1.94 cm^2^/g at 100 keV), bones (0.186 cm^2^/g at 100 keV), and soft tissues (0.169 cm^2^/g at 100 keV). The K-edge of Au (80.7 keV) confers higher absorption than iodine (33.2 keV) [51]. Au has a higher X-ray absorption than iodine at around 100 keV and at low energies (< 30 keV), which is a useful energy range for clinical CT, fluoroscopy, and mammography. Previous studies on high-atomic-number agents indicate that good contrast images can be obtained at a concentration of 100 μg Au/mL [52]. This level is approximately 100 times lower than the toxic dose of Au. Small amounts of Au clinically improve the safety margin and reduce the cost. 

Compared with iodine-based contrasts, the absorbance of Au is three times higher at 100 keV while the density is five times higher (1.5 g Au/cm^3^* vs.* 0.3 g iodine/cm^3^). Therefore, the overall contrast gain is greater than 10-fold. Therefore, improved visualization can be achieved using AuNP contrast media compared to iopromide. At a concentration of 0.5077 M, AuNP contrast enhancement is up to 88% greater than iodine at low energies and up to 115% greater at high energies according to signal-to-noise ratio [53]. There is no significant improvement in contrast compared to iodine at moderate tube potentials (70–90 kVp). Alternatively, comparable enhancement levels should be possible with lower molar concentrations of Au compared to iodine. With this important usefulness, AuNP applications in X-ray imaging are discussed in the next section with recent research examples.

## 6. Gold Nanoparticles (AuNPs) in X-ray Imaging

### 6.1. Properties and Applications of AuNPs

Metal nanoparticles (NPs) have been used in extensive biomedical applications because of their small size, high thermal stability. 

Among metal NPs, AuNPs are a prominent choice because of their amenability for synthesis, functionalization and detection as well as low toxicity. AuNPs with controlled geometrical, optical, and surface chemical properties have been intensively studied for practical applications in biology and medicine. Versatile functionalization is one of the important advantages of AuNPs and it facilitates the targeted delivery of AuNPs to various types of cells, bioimaging, gene/drug delivery, as well as other therapeutic and diagnostic applications [54]. The useful categories of the AuNP functionalization and their applications are summarized in Table 2.

**Table 2:** Summary of common functionalization of AuNPs and their applications [54].

Functional Group	Ligands/Carrier Molecule	Key Feature	Application	References
Polyethylene Glycol (PEG)	PEG with ligands such as a dye attached through thiol group	Adherence to the cell membrane	Cellular and intracellular targeting, biodistribution studies	[55,56,57,58,59]
Amine Group	PEG	siRNA carrier	Useful in RNAi technology	[60]
Carboxyl Group	Proteins		Various depending on the protein	[61]
Peptide	Cell surface receptors, amyloid inhibitory peptide + sweet arrow peptide, antibody, octrotide peptide	Cytoplasmic and nuclear translocation, adjuvant, targeting carcinoma cells analogue of somatostatin	Cellular and intracellular targeting, macrophage and proinflammatory cytokine elicitation bioimaging imaging of cancer cells	[62,63,64,65,66]
DNA	Aptamer, PEGylated goldpoly (β-amino ester), Thiolated ssDNA of RNA I gene, antisense DNA oligonucleotides	Targeting Prostate cancer cells, siRNA carrier, binds to antisense RNA	Bioimaging, gene delivery rnai-regulation of transgene expression, detection of specific genes e.g., for microbial detection	[67,68,69,70,71]
RNA	Polyvalent RNA-goldnanoconjugates		RNAi	[72]
Antibodies	scFv Antibodies against various pathogens	Smaller size, label fidelity	Immunoassays treatment and diagnosis e.g., antibodies against aflatoxins	[73,74]

**Figure 4:**
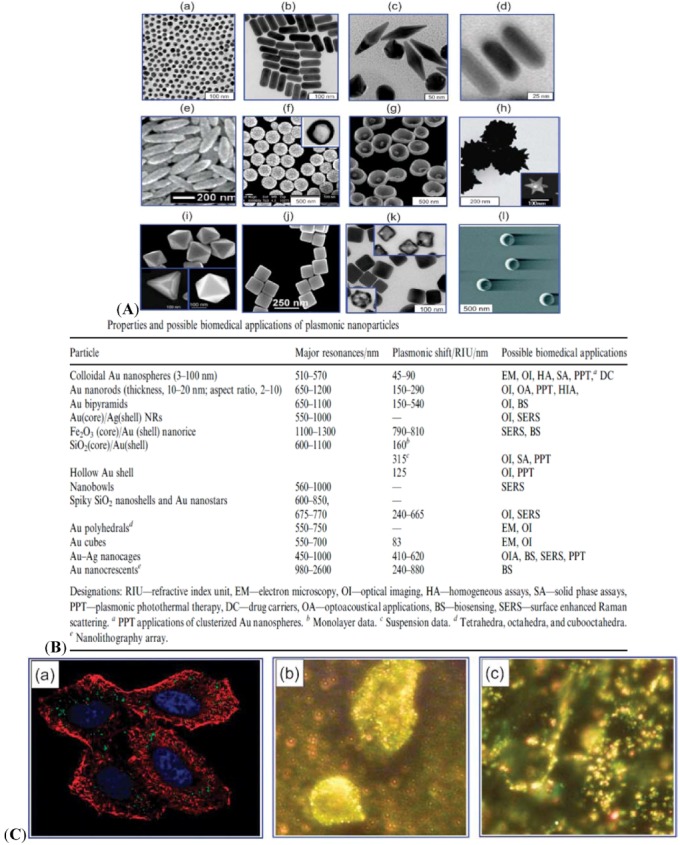
(**A**) Various types of plasmon-resonant nanoparticles: 16 nm nanospheres (**a**) Au nanorods (**b**) Au bipyramids (**c**) Au nanorods surrounded by silver nanoshells (**d**) nanorice (Au-coated Fe_2_O_3_ nanorods) (**e**) SiO_2_/Au nanoshells (**f**) (the inset shows a hollow nanoshell); nanobowls with bottom cores (**g**) spiky SiO_2_/Au nanoshells (**h**) (the inset shows a Au nanostar); Au tetrahedra, octahedra, and cubooctahedra **(i)** Au nanocubes (**j**) Ag nanocubes and Au-Ag nanocages (obtained from those in the insets) (**k**) as well as Au nanocrescents (**l**) [9]. (**B**) Properties and possible biomedical applications of plasmonic nanoparticles. (**C**) Confocal image of HeLa cells in the presence of AuNPs [77]. (**a**) Blue indicates the nuclei stained with Hoechst 33258. Red indicates the actin cytoskeleton labeled with Alexa Fluor 488 phalloidin. Green indicates unlabeled AuNPs. The image is taken by two-photon microscopy. Dark-field microscopy of cancerous (**b**) and healthy (**c**) cells using AuNPs conjugated with antibodies for epidermal growth factor [78]. Adapted from the data of the cited papers by permission from The Royal Society of Chemistry, and The American Chemical Society.

Au is employed in various bioimaging methods [75,76]. One prominent property of Au nanomaterial is plasmonics, which enables broad biomedical applications. The various shapes of Au nanomaterials are illustrated in Figure 4A [9]. 

In addition to the typical spheres, nanorods, nanoshells, nanocages, nanostars, and other types have been developed. Figure 4B shows a summary of shape-dependent plasmonic properties and possible biomedical applications [9]. Recently, optical microscope technologies such as confocal or multi-photon microscopy have been popularly used to visualize AuNPs. These technologies enable the luminescence of AuNPs excited by the simultaneous absorption of multiple photons. Confocal images of AuNP-incorporated HeLa cells are shown in Figure 4C(a) [77]. Dark-field microscopy is another popular bioimaging method based on the light-scattering property of microscopic objects as exampled in Figure 4C(b) and Figure 4C(c) [78]. In this method, AuNPs can more accurately reveal bio-specific interactions than other fluorescence methods. 

Au is easily formed into various sizes and shapes with versatile functionalities [1,9,54]. However, the X-ray imaging results indicate that contrast enhancement is less dependent on size and shape of the nanoparticles [79]. AuNPs are useful in various biosensing applications [2], such as cancers [3], as a biocompatible imaging agent [4]. Intravenously injected AuNPs are effectively accumulated in organs, demonstrating possible applications of Au in the early diagnosis and medical treatment of vascular diseases. The extended imaging time and high contrast provided by AuNPs are useful for many biomedical applications, including non-invasive imaging of blood flows in coronary and cerebral arteries, assessment of atherosclerotic plaque/stenoses, delineation of stroke/arteriovenous malformation/aneurysms, determination of vascularity, and enhancement of mammography/renal angiography/virtual colonoscopy. Improved contrast for better prognoses possibly enables the non-invasive detection of small tumors (e.g., <1 cm) which are missed by current available techniques [49]. Tumor vascularity is closely related with invasiveness [50], thus vascularity indices make non-invasive staging possible. AuNPs can also distinguish highly vascularized vulnerable plaques from stable plaques. 

### 6.2. AuNP-Incorporated Microparticles as an X-ray Imaging Contrast Agent

With the advent of faster CT machines, the Au-enhanced imaging of coronary arteries, particularly in obese patients or those with mural calcifications has become feasible via transvenous injection of AuNPs without resorting to arterial catheterization. Because the concentration of Au can be made five times higher than that of iodine agents, transarterial catheterization using AuNP is also beneficial, particularly for large patients who require additional contrast. Extravasation is evident from the extensive retention of AuNPs in tumors after transvenous injection, compared with a much lower accumulation in muscles. This retention of AuNPs is useful for enhanced tumor detection and chemical therapy. 

**Figure 5:**
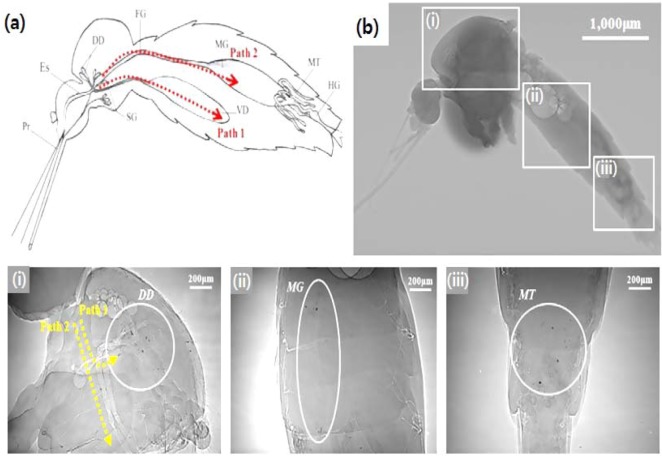
(**a**) General anatomical diagram of a mosquito. The dorsal diverticulum [DD], foregut [FG], hindgut [HG], midgut [MD], malpighian tubules [MT], esophagus [ES], proboscis [Pr], salivary glands [SG], and ventral diverticulum [VD] are indicated. (**b**) Synchrotron X-ray images of a female mosquito that has taken up the AuNP-incorporated chitosan microparticles. A white beam X-ray source (8–30 keV) is utilized to maximize the absorption capability of the designed X-ray contrast flow tracer. The images are captured at 5 h after the consumption of the microparticles. Scale bar 1000 μm in the side view of the mosquito. The scale bar is 200 μm for three magnified images (i, ii, and iii). The three white boxes indicate the thorax (i), upper abdomen (ii), and hypogastrium (iii) of a mosquito. (i) Side, (ii and iii) front views of the mosquito. Paths 1 and 2 in (i) indicate the two digestive routes specialized for the different foods consumption of a mosquito. The locations of the particles are highlighted by circles [81]. Adapted by permission from Elsevier.

**Figure 6:**
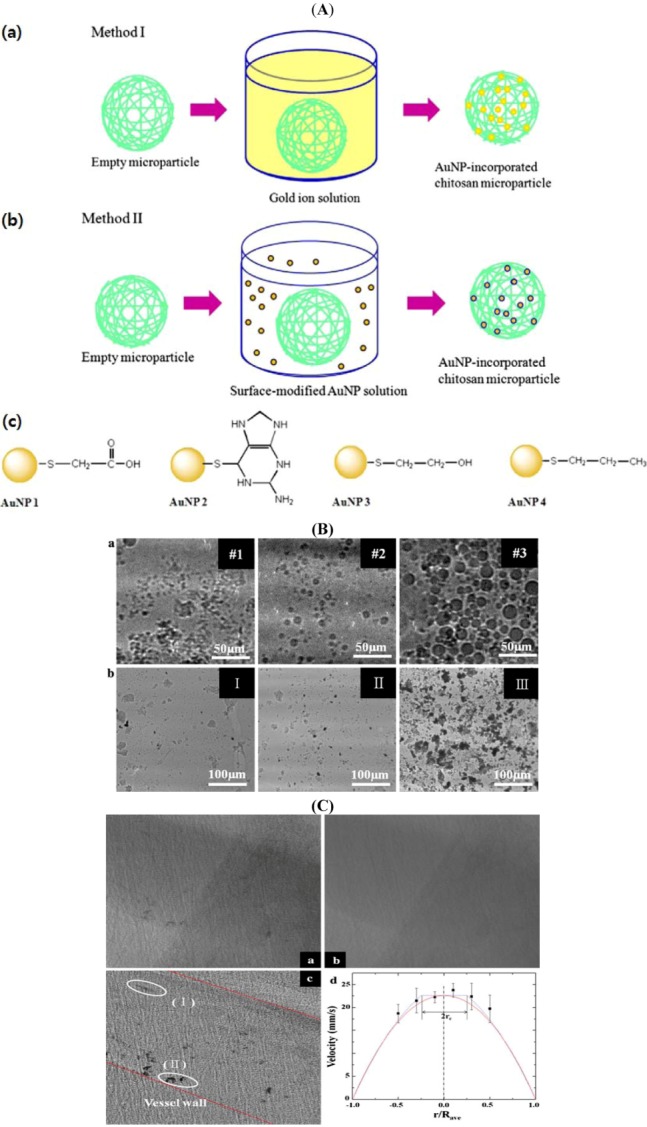
(**A**) AuNP incorporation into chitosan microparticles. (**a**) The Au ions are reduced in the empty microparticles (Method I). (**b**) Surface-modified AuNPs diffuse into the empty microparticles (Method II). (**c**) Surface-modified AuNPs used in Method II [83]. (**B**) (**a**) X-ray images of the dried microparticles fabricated by Method I (Figure 5B) with 6.35 × 10^−2^ mmol Au ions. The size of the microparticles increases according to the molecular weight of chitosan: #1 (20,000 Da), #2 (30,000 Da) and #3 (85,000 Da). (**b**) X-ray images of the microparticles containing reduced AuNPs. AuNPs are reduced in the chitosan microparticles (20,000 Da) at 6.35 × 10^−3^ [I], 3.15 × 10^−2^ [II], and 6.35 × 10^−2^ [III] mmol Au [60]. **(C**) A typical raw (**a**), averaged (**b**), and preprocessed (**c**) X-ray image of the blood flow in a rat cranial vena cava. (**d**) The velocity vectors obtained from the rat vein. The dotted line represents the velocity profile of the blood flows as suggested by Casson model. The solid line denotes the parabolic curve fitting on the experimental data. R_ave_ is the vessel radius averaged from X-ray images [6]. Adapted by permission from Springer.

Recently, AuNPs were employed in various research areas as a unique contrast-enhancing X-ray flow tracer. For example, they are employed to track the dynamic sap flow in plant vessels [80]. AuNPs are incorporated into a polymeric microparticle as an X-ray contrast flow tracer designed for particle tracing analysis in a living insect [81]. Figure 5 illustrates the AuNP-incorporated polymeric (chitosan) microparticles utilized in tracking the digestive pathways of live female mosquitoes. The images are obtained by the synchrotron X-ray. The average size range of the designed tracer microparticles is between 10 and 20 μm depending on the fabrication methods (Figure 6A). This size range is designed to be analogous to the size of blood cells in humans. In addition, the feature size of tracer microparticles containing AuNPs is important for optimal spatial resolution to overcome the errors encountered in dynamic X-ray imaging analysis. The X-ray imaging efficiency of X-ray contrast agent-incorporated polymeric microparticles are systematically discussed in terms of Beer-Lambert Law [48,82]. 

Chitosan is an effective reducing agent of Au^3+^ ion and simultaneously an effective stabilizer for the formed AuNPs. For high contrast in the X-ray images, the loading efficiencies of AuNPs in chitosan microparticles are controlled. Figure 6A illustrates the two methods to incorporate AuNPs in the polymeric microparticles. In the first method, a controlled amount of Au^3+^ ion is dissolved in Milli-Q DI water, modulating the Au concentration in solution. Empty microparticles are fabricated by cross-linked chitosan polymers by a microemulsion template. The concentration-controlled Au^3+^ ion aqueous solution is mixed with the empty microparticles [Method I in Figure 6A(a)]. The mixed solution is then heated until the solution turns from yellow to deep purple, indicating AuNP formation by Au^3+^ ion reduction in the microparticles. In the second method [Method II in Figure 6A(b)], surface-modified AuNPs in water are prepared with a fixed number density. These concentration-controlled surface-modified AuNP solutions are physically mixed with the aforementioned empty microparticle solutions. Depending on the surface modification [Figure 6A(c)], the AuNP incorporation efficiency into empty microparticles is differentiated. 

Figure 6B (a, top low) shows the X-ray images of the AuNP-incorporated microparticles fabricated by Method I in Figure 6A. The size of the microparticles increases by the molecular weight of chitosan: #1 (20,000 Da), #2 (30,000 Da) and #3 (85,000 Da) at a fixed 6.35 × 10^−2^ mmol Au^3+^ ion solution [6,50]. The Beer–Lambert law is employed to evaluate the absorption coefficient of the designed AuNP-incorporated chitosan microparticles in the synchrotron X-ray imaging [48,82]. The absorption coefficient decreases with increased molecular weight of chitosan (#1 (475.21 μm^−1^) > #2 (417.91 μm^−1^) > #3 (274.85 μm^−1^)) [6,81]. Figure 5C (b, bottom low) shows the typical X-ray images of the cross-linked-microparticles formed by concentration-controlled Au^3+^ reduction. Empty chitosan particle (20,000 Da) is treated with 6.35 × 10^−3^ mmol (I), 3.15 × 10^−2^ mmol (II), and 6.35 × 10^−2^ mmol (III) of Au^3+^ ion solution. As the Au^3+^ ion concentration increases, the imaging efficiency is gradually improved because of the increased efficiency of AuNP loading.

Figure 6C(a) shows a synchrotron X-ray image of blood flow in the cranial vena cava of a rat. One hundred images are averaged, and then the averaged images are subtracted from the number of raw images to remove background noises generated by the surrounding tissues [Figure 6C(b)]. 

Thereafter, the intensity range is readjusted to make the tracer particles more distinct. Compared with the raw images, the individual AuNP–chitosan particles (I) and their clusters (II) are observed in the rat vein with high image contrast [Figure 6C(c)]. In Figure 6C(d), velocity profiles are analyzed. The average diameter, *D*_ave_ of the blood vessel is 652.42 μm. The particle streak formed by particle cluster movement is measured by assuming that the blood vessel wall is a linear line. Each particle cluster is distinguishable, and most clusters are located near the bottom wall, 0.157*D*_ave_ of the vein by the gravity. The individual particles appear as thread-like streaks because the flow speed is fast compared with the exposure time of X-ray illumination (10 ms). The particle velocity is obtained by dividing the length of each streak by the exposure time. Although individual particles appear as thread-like streaks, this is the first trial to measure *in vivo* blood flow in a live animal dynamically using X-ray particle image velocimetry technique. This study contributes to the basic understanding on the fluid mechanics of circulatory vascular diseases.

### 6.3. Gold Nanoparticle (AuNP) Contrast Agents in X-ray Imaging

**Figure 7:**
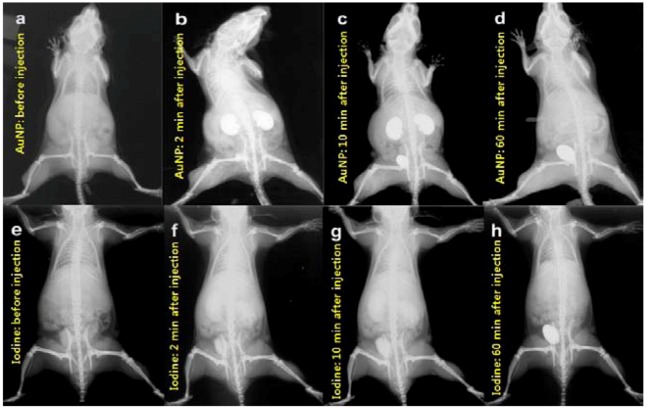
Pharmacokinetics of AuNPs (**a**–**d**) and iodine contrast agent (**e**–**h**) in mice. (**a**,**e**) Before injection. (**b**,**f**) 2 min after injection; (**c**,**g**) 10 min after injection; (**d**,**h**) 60 min after injection. The AuNPs show low liver and spleen uptake as well as clearance via kidneys and bladder (**b**–**d**). At 60 min (**d**), the contrast in the Au-injected mouse is similar to the uninjected mouse (**a**), indicating efficient clearance. A Lorad Medical Systems mammography unit (Hologic, Inc., Danbury, CT, USA; model XDA101827) is used with 8 mAs exposures (0.4 s at 22 kVp). Kodak Min-R2000 mammography film, 18 cm × 24 cm (Eastman Kodak, Rochester, NY, USA) is used [50]. Adapted by permission from The British Institute of Radiology.

AuNPs of 1.9 nm in diameter are suspended in a phosphate-buffered saline (PBS) and injected via a tail vein into Balb/C mice bearing EMT-6 subcutaneous mammary tumors (Figure 7) [50]. The AuNP enables direct imaging, detection, as well as measurement of angiogenic and hypervascularized regions. Images taken at different times after intravenous injection show that the small AuNPs are not concentrated in the liver and the spleen, but are cleared-out through the kidneys. These animal studies demonstrate that AuNPs are useful X-ray contrast agents, offering novel physical and pharmacokinetic advantages over typical I odine-based agents. They are non-toxic, provide higher contrast, and enable longer imaging times than standard Iodine-based agents.

#### 6.3.1. Biomedical Applications of AuNPs

**Figure 8:**
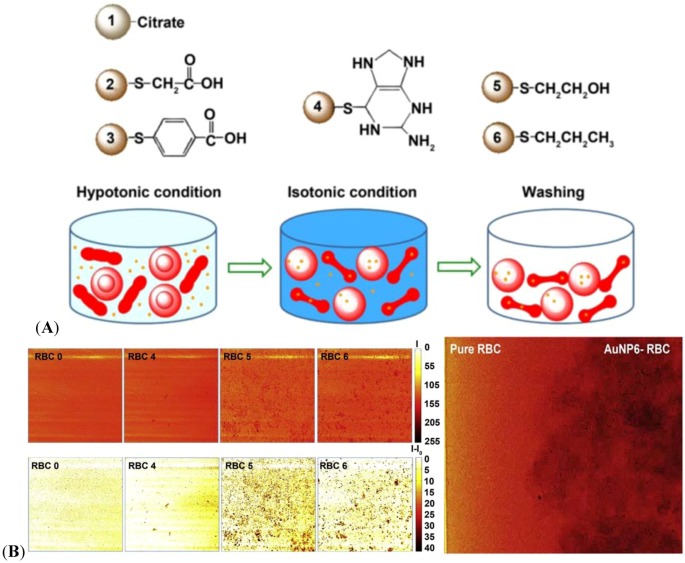
(**A**) Six types of AuNPs of 20 nm diameter are designed for incorporation into RBCs. The AuNPs are reduced by sodium citrate tribasic dihydrate (AuNP 1). For surface-modification, each AuNP are covered with thioglycolic acid (SH-CH_2_COOH) (AuNP 2), 4-mercaptobenzoic acid (SH-Ph-COOH) (AuNP 3), 6-thioguanine (SH-C_5_H_4_N_5_) (AuNP 4), 2-mercaptoethanol (SH-CH_2_CH_2_OH) (AuNP 5), and 1-propanthiol (SH-CH_2_CH_2_CH_3_) (AuNP 6) [83]. (**B**) (left) X-ray images of selected RBC 0 (without AuNP incorporation), RBC 4 (AuNP 4-incorporated RBC), RBC 5 (AuNP 5-incorporated RBC), and RBC 6 (AuNP 6-incorporated RBC) are detected by the enhanced phase contrast together with enhanced absorption contrast. The upper images are flat-field-correction images expressed by the absolute X-ray absorption (I). The lower images show the X-ray absorption intensities subtracted by the corresponding background (I–I_0_). The scale bars indicate the degree of absorption. The size of the images is 500 mm × 500 mm. A short video of X-ray images capturing the time-dependent flow motion of RBC 6 is available in Ref. [63]. Adapted by permission from Elsevier.

Time-resolved dynamic imaging of bio-fluids provides valuable information for the clinical diagnosis and treatment of circulatory disorders. For quantitative information on non-transparent blood flows, particle-tracing dynamic X-ray imaging is employed which needs better spatial resolution and more enhanced image contrast compared with static imaging. Therefore, tracer particles tagging the flow streams are critical. Taking advantage of the high X-ray absorption, AuNPs are incorporated into the human RBCs as shown in Figure 8A [83]. RBC is an advantageous blood flow tracer because it is the natural and primary component of blood. The loading efficiency of AuNPs into RBC is critical to the enhancement of the imaging contrast. Depending on the surface properties of the AuNPs (Figure 8A), the AuNP-incorporated RBC provides a diverse contrast, as shown in Figure 8B. Both hydrophilically (AuNP 5-incorporated RBC, RBC 5) and hydrophobically (AuNP 6-incorporated RBC, RBC 6) surface-modified AuNPs are found to be effective. The enhanced dynamic X-ray imaging of blood flows can be used for clinical applications in the future as tested in Figure 8B, right.

X-ray-based CT is one of the most convenient imaging/diagnostic tools in hospitals today in terms of availability, efficiency and cost. However, in contrast to MRI and other nuclear medicine imaging modalities, CT is not considered as a molecular imaging modality because targeted and molecularly specific contrast agents have not yet been developed. A targeted molecular imaging platform that enables cancer detection at the cellular and molecular levels with standard clinical CT has been introduced recently [84].

This method is based on AuNPs which sensitively target tumors with selective antigens while inducing a distinct contrast in X-ray CT imaging. An *in vitro* demonstration of head and neck cancer imaging shows that the attenuation coefficient for the molecularly targeted cells is more than five times higher than the image of untargeted cancer cells or normal cells. This novel imaging technology can lead to significant improvements in cancer therapy in terms of earlier detection, accurate staging, and microtumor identification. A new imaging technique that utilizes surface-modified AuNPs in combination with X-ray imaging has been developed for the early diagnosis of hepatocellular carcinoma [8]. The tissues labeled with these electron-dense particles exhibit enhanced X-ray scattering over normal tissues, distinguishing the cells containing AuNPs from those without AuNPs in the X-ray scatter images. This novel approach enables the *in vivo* detection of tumors, as small as a few mm in size.

**Figure 9:**
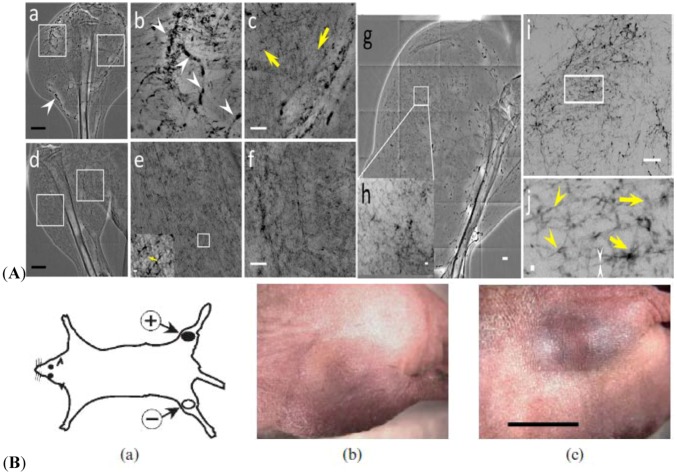
(**A**) X-ray micrographs of the microvasculature of normal tissue and tumors at different time period after the tumor inoculation with AuNPs and heparin injection. Micro-radiology is implemented with unmonochromated (white) synchrotron X-rays.(**a**) *In vivo* image of the lateral thigh, 7 days after inoculation. (**b** and **c**) Magnified images of the left square in (**a**), near the tumor area, and the right square, which correspond to normal tissue area (medial thigh). The arrowheads in (**a**) and (**b**) mark vessels showing AuNP agglomeration. The yellow arrows mark vessels of < 6 μm diameter. (**d**) *In vivo* image of the normal lateral thigh. (**e**, **f**) Magnified images of the left and right squares in (**d**). The inset in the lower left corner of (e) is also magnified image. The yellow arrow marks a < 6 μm diameter vessel. (**g**) *In vivo* image of the lateral thigh, 16 days after inoculation. (**h**) Magnified image of the square in (**g**). (**i**) Image of a 1 mm thick tissue removed from the thigh shown in (**g**). (**j**) Magnified image of the rectangle in (**i**). The yellow arrowheads mark abnormal vessels. The white arrowheads mark a vessel with ~2 μm diameter. The yellow arrows show areas with diffusion of AuNPs. Scale bar for (**a**), (**d**) and (**g**): 2 mm. Scale bar for (**b**), (**c**), (**e**), (**f**), and (**i**): 500 μm. Scale bar for inset of (**e**) and (**h**): 50 μm. Scale bar for (**j**): 10 μm. [58]. (**a**) Mouse model with two implanted tumors: Her2+ in one thigh and Her2– in the contralateral thigh. (**b**, **c**) The same mouse with a Her2– tumor (**b**) and Her2+ tumor (**c**) 20 h after intravenous injection of 15 nm AuNP–Herceptin (0.45 g Au kg^−1^ body weight). Tumor volumes (by caliper measurements) are nearly identical, but a distinction can be seen owing to the targeted Au localization. Bar = 55 mm [66]. Adapted by permission from BioMed Central and The British Institute of Radiology.

Angiogenesis has been widely investigated in conjunction with cancer proliferation, and particularly for the possibility of early-stage detection and new therapeutic strategies [85]. However, these studies are hindered by the limitations of imaging techniques in the detection of microscopic blood vessels of 3–5 μm in diameters, grown under angiogenic stress. Synchrotron X-ray imaging techniques with suitable contrast agents can overcome this obstacle with high spatial resolution. AuNPs are tested to detect cancer-related angiogenesis by synchrotron microradiology, microtomography, and high-resolution X-ray microscopy. Figure 9A shows that AuNP injection provides sufficient contrast to allow the *in vivo* detection of small capillary species (the smallest measurable lumen diameters are 3–5 μm). With the AuNPs mixed with heparin, local extravascular AuNP diffusion in tumor areas is detected where capillary leakage occurs. Although AuNPs are superior radiology contrast agent candidates, their success is not guaranteed, particularly in targeting very small blood vessels in tumor-related angiography. However, AuNPs produce sufficient contrast to observe tumor microvessels of 3–5 μm diameters, as well as extravascular diffusion because of basal membrane defenestration. These results demonstrate the possibility of functional imaging of the tumor microvasculature and the effects of anti-angiogenic drug effects in X-ray imaging.

AuNPs are potential *in vivo* diagnostic and therapeutic agents, X-ray contrast agents, drug delivery vehicles, as well as radiation enhancers. The targeting and microlocalization of AuNPs are quantitatively determined by micro-CT after intravenous injection in mouse tumor models [86]. After intravenous injection of 15 nm AuNPs coupled with herceptin into nude mice, simultaneously bearing subcutaneous tumors in the opposite thighs of a mouse [Figure 9B(a)], specific AuNP accumulation is detected visually [Figures 9B(b) and (c)] in the tumors. The mouse model has two implanted tumors, Her2– in one thigh (b) and Her2+ in the contralateral thigh (c). The same mouse with a Her2–(b) and Her2+ (c) 20 h after 15 nm AuNP-herceptin intravenous injection (0.45 g Au kg^−1^ body weight). 

The tumor volumes determined by caliper measurements are nearly identical, but a distinction based on the targeted Au localization can be observed. Although some AuNPs appear non-toxic at useful levels [45,75], more testing is needed. Another advantage is the prolonged tumor contrasting (20 h in this study) compared with the more ephemeral MRI and PET contrast imaging. Knowing the exact tumor topology enables proper surgery, radiation treatment, monitoring, and other image-guided procedures. Imaging provides a potential alternative to painful and invasive biopsies that may not accurately sample the tumor. A serious problem with current surgery and radiotherapy is that it is difficult to exactly determine the true tumor boundaries.

#### 6.3.2. Dynamic Information Obtained by AuNP-Contrasted X-ray Imaging in Biological Systems

**Figure 10:**
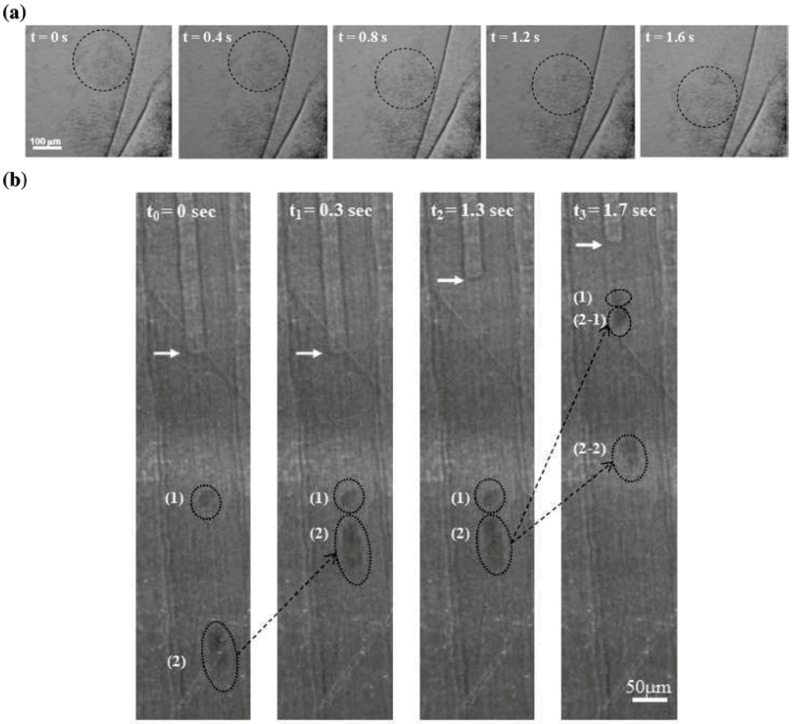
Application of AuNPs to the visualization of fluid dynamics in a living organism. (**a**) Time-dependent flow motions of the concentration-controlled hydrophilic AuNP aqueous solution captured by synchrotron X-ray imaging. (**b**) Four consecutive images showing the uptake and transport of hydrophilic AuNPs inside the xylem vessels of a rice leaf sheath visualized by synchrotron X-ray imaging. The arrow indicates the meniscus between water and air. (1) and (2) are initial positions of hydrophilic AuNP clusters in the sap. At *t*_0_ = 0 s and *t*_0_ = 0.3 s, only cluster (2) moves upward. At *t*_0_ = 1.3 s and *t*_0_ = 1.7 s, cluster (1) moves up, cluster (2) is simultaneously separated into two distinct clusters (2−1) and (2−2) with different velocity. The images are captured consecutively by the synchrotron X-ray source using a bending magnet. X-rays with a peak energy of 20.3 keV (8–30 keV range) are applied as a function of time without monochromator to obtain high energy [80]. Adapted by permission from The American Chemical Society.

Figure 10(a) displays the time-dependent flow motion of the concentration-controlled hydrophilically surface-modified AuNPs in water caused by gravity [80]. The images are consecutively captured by synchrotron X-ray at a speed of 25 frames per second. Time-resolved images are captured through the Kapton window covering a sample holder during the designed time. When the concentration of AuNP increases to 2.4 × 10^19^ AuNPs/m^3^, the dynamic images of AuNP movement are obtained.

In contrast to many-reported static accumulation of contrast agents in targeted biological organs, studies on the dynamic motion of biomaterials in a living organism are rare. The penetration and movement of magnetic iron−carbon NPs in *Cucurbita pepo* cells have been analyzed [87]. NPs with diameters larger than 50 nm cannot be detected inside a plant tissue, indicating the importance of a size-based filtering mechanism. Flow phenomena of xylem vessels in rice are monitored by synchrotron X-ray imaging using AuNPs of 20 nm in diameter as a flow-tracing sensor [80]. Adopted from the *in vitro* condition in Figure 10(a), the concentration of 2.4 × 10^19^ AuNPs/m^3^ is successful to obtain clear flow images even inside the xylem vessels of live rice. 

This methodology is developed to utilize the surface-modified AuNPs (in this case, hydrophilically surface-modified AuNP in water) as particulate flow tracers in visualizing the dynamic uptake and transport of sap flow in the xylem vessels of a plant. In Figure 10(b), the spots of (**1**) and (**2**) represent the displacement of the hydrophilic AuNPs at the beginning (t = 0 s). As the flow continues (t = 0.3, 1.3 and 1.7 s), the AuNP positions are diversified by the balance between the resistance produced by perforated plates crossing the xylem vessels and the pulling capacity. The motion of these AuNPs is not identical to the movement of the air/water meniscus indicated by the white arrows at the upper part of the xylem vessels. A sudden uptake controlled by the speed of the hydrophilic AuNP movement occurs, whereas the upper meniscus is directly stopped at the location of the perforated plate in the xylem vessel. This movement explains the existence of radial water transport to adjacent xylem vessels. This kind of *in vivo* monitoring provides new insights into the visualization of axial and radial sap flows in plant xylem vessels in detail. Conventional imaging methods without suitable flow tracers cannot successfully visualize these flow phenomena. For the broad basic sciences of biological systems, X-ray imaging with high penetration and high resolution are advantageous, especially when combined with a suitable X-ray contrast agent such as AuNP. 

### 6.4. Renal Clearance and Toxicity of AuNPs

Efficient renal clearance and minimum accumulation/interference of nanomaterials employed for *in vivo* use are fundamentally important [88]. *In vivo* applications of many nanomaterials designed for drug delivery, biomedical imaging, and photothermal therapies are still limited by their slow renal clearance and non-specific accumulation in the organs of the reticuloendothelial system (RES), such as liver and spleen [89]. Although NPs with hydrodynamic diameters smaller than 10 nm are generally stealthy to the RES organs, these NPs are still found in the liver [90]. For example, nearly 50% of 1.4 nm AuNPs are found in the liver, and only about 9% of them can be excreted via urine within 24 h after intravenous injection [91]. Nonetheless, the renal clearance of AuNPs is improved by surface modification. For example, compared with similarly-sized AuNPs coated with cysteine, glutathione-coated AuNPs (GS-AuNPs) can significantly enhance renal clearance *in vivo* [5]. Approximately 2 nm GS-AuNPs display 3.7−1.9% of the accumulation in the liver and more than 50% of GS-AuNPs are found in urine within 24 h after intravenous injection, which is comparable to one of the best renal clearance efficiencies obtained with quantum dots.

Although Au itself is believed to be biocompatible and has long been used for biomedical applications, many studies have been conducted to evaluate the actual use of AuNPs *in vivo*. Most data show that the surface chemistry and physical dimensions of AuNPs play important roles in toxicity. *In vivo* toxicity of PEG-coated AuNPs in mice has been investigated in this point of view [92]. Size-dependent toxicity has been studied with naked AuNPs without surface-modification ranging from 3 to 100 nm injected intraperitoneally into BALB/C mice at a dose of 8 mg/kg/week [93]. AuNPs ranging from 8 to 37 nm induce severe sickness in mice such as fatigue, loss of appetite, fur color change, and weight loss. Injection of 5 and 3 nm AuNPs, however, did not induce sickness or lethality in mice. 

The dose effect (40, 200, and 400 μg/kg/day of AuNPs) also has been investigated by evaluating the bioaccumulation and toxic effects. AuNPs with a fixed-size of 12.5 nm are administered upon intraperitoneally in mice every day for 8 days [94]. The result shows that the Au levels in blood do not increase with the administered dose. On the other hand, all examined organs show a proportional increase with the Au level, indicating efficient tissue uptake. No evidence of toxicity is observed in any of the diverse studies performed, including survival, behavior, animal weight, organ morphology, blood biochemistry and tissue histology. Thus, the AuNP accumulation pattern in tissue depends on the administered doses but does not inflict subacute physiological damage.

### 6.5. Cost of AuNPs

Currently, NP fabrication itself requires an elaborate procedure, which leads to relatively high cost for many materials such as Au, Bi, Fe, W, Mo, Cr, Al, Mg, Mn, Ti, Sn, Co, Ag, Zn, Fe, Ni, Si, Al, Tl, Cu, and others with size ranges less than 100 nm. The ability of NPs for improved target properties has thus been commercially limited by the high cost of production. Technologies have been developed to enable more cost- and energy-efficient NP production. In the same vein, various technologies have been developed to decrease the AuNP production cost. Quantitative chemical applications of metal NPs require colloidal samples containing a large number of pure and well-defined particles with high monodispersity. An ideal separation method for the size selective purification of metal colloids should be fast and cost-effective, providing a sufficient size resolution of 1–250 nm. Electrophoresis, for example, has been successfully applied to separate particles with different size and shape [95]. Currently, the production of NPs generally falls into three categories: solid phase particle reduction, liquid phase synthesis, and gas phase synthesis. In addition to economic considerations, each process suffers from limitations ranging from poor property control to the introduction of external contaminants into the product. To overcome these limitations, new technologies such as plasma reaction processes have been developed to produce low-cost and high-performance NPs from inexpensive solid feed-stocks [96]. Specifically, this research evaluates and optimizes a modular hybrid plasma reactor to produce nanomaterials with consistent particle morphology and size (>95% nanomaterials less than 100 nm), desirable surface chemistry, and reduced contaminants (>99.5% purity). The plasma reactor system must be able to run continuously with feed materials for a minimum of 8 hours, which is several times longer than current technologies.

## 7. Conclusions

In this review we have selected some examples that illustrate the substantial progress utilizing AuNPs in X-ray imaging. In addition to the typical biomedical imaging, the applications are growing rapidly as synchrotron radiation facilities which provide a variety of advanced X-ray imaging techniques appear around the World. Furthermore, the range of scientific questions that can be addressed is increasing as X-ray imaging capabilities improve. Continued progress in the development of AuNP-based X-ray imaging will support the participation of a wider range of academic, biomedical and industrial researchers, and the emergence of intense, wide-ranged energy sources will open completely new opportunities for dynamical studies in the realm of nanometer science. 

X-ray imaging is one of the most popular imaging methods and has great potential to become highly powerful in many future applications. The general direction of the development of AuNP-based X-ray imaging technologies is toward achieving higher spatial resolution, smaller-sized imaging, fast time-resolved imaging, and a variety of practical applications. Fundamentally, single cell-based and single molecule-based imaging technologies are required in many research areas. Combined with drug/imaging agent delivery and NP-mediated disease treatment AuNP-based X-ray imaging has strong potential in biomedical applications, which facilitates rapid expansion of bioscience researches. Utilizing the characteristic interaction between a biological system and AuNPs, X-ray imaging has a unique potential to reveal still unknown variety of scientific phenomena. Therefore, AuNP-based X-ray imaging greatly contributes to the wide ranges of bioscientific and biotechnological studies that are currently being developed and expected to be developed in the future.
